# Environmentally persistent free radicals induce airway hyperresponsiveness in neonatal rat lungs

**DOI:** 10.1186/1743-8977-8-11

**Published:** 2011-03-09

**Authors:** Shrilatha Balakrishna, Jordy Saravia, Paul Thevenot, Terry Ahlert, Slawo Lominiki, Barry Dellinger, Stephania A Cormier

**Affiliations:** 1 Department of Pharmacology and Experimental Therapeutics, Louisiana State University Health Sciences Center, New Orleans, Louisiana, USA; 2 Department of Chemistry, Louisiana State University, Baton Rouge, Louisiana, USA; 3 Department of Pharmacology, Yale University School of Medicine, New Haven, CT, USA

## Abstract

**Background:**

Increased asthma risk/exacerbation in children and infants is associated with exposure to elevated levels of ultrafine particulate matter (PM). The presence of a newly realized class of pollutants, environmentally persistent free radicals (EPFRs), in PM from combustion sources suggests a potentially unrecognized risk factor for the development and/or exacerbation of asthma.

**Methods:**

Neonatal rats (7-days of age) were exposed to EPFR-containing combustion generated ultrafine particles (CGUFP), non-EPFR containing CGUFP, or air for 20 minutes per day for one week. Pulmonary function was assessed in exposed rats and age matched controls. Lavage fluid was isolated and assayed for cellularity and cytokines and *in vivo *indicators of oxidative stress. Pulmonary histopathology and characterization of differential protein expression in lung homogenates was also performed.

**Results:**

Neonates exposed to EPFR-containing CGUFP developed significant pulmonary inflammation, and airway hyperreactivity. This correlated with increased levels of oxidative stress in the lungs. Using differential two-dimensional electrophoresis, we identified 16 differentially expressed proteins between control and CGUFP exposed groups. In the rats exposed to EPFR-containing CGUFP; peroxiredoxin-6, cofilin1, and annexin A8 were upregulated.

**Conclusions:**

Exposure of neonates to EPFR-containing CGUFP induced pulmonary oxidative stress and lung dysfunction. This correlated with alterations in the expression of various proteins associated with the response to oxidative stress and the regulation of glucocorticoid receptor translocation in T lymphocytes.

## Background

There is little doubt that exposure to airborne particulate matter (PM) poses a significant health risk, and there is strong evidence to support the basic concept that fine and ultrafine PM exposure have adverse pulmonary effects. Increased amounts of ambient PM have been associated with asthma and chronic obstructive pulmonary disease (COPD) exacerbations, increased hospitalizations for respiratory diseases, lung function decline, and even increased respiratory mortality in susceptible populations, including infants and children [1-7]. Despite the significant epidemiological evidence demonstrating an association between PM exposure and adverse pulmonary effects, the mechanisms responsible for the adverse pulmonary effects are not entirely clear. Moreover, few experimental studies using age-relevant animal models have been used in order to investigate the detrimental effects of PM on developing lung function.

Airborne PM is a complex mixture of chemical species, and the unique components in PM that are responsible for adverse health effects remain elusive. A number of anthropogenic sources including combustion processes generate PM. These emissions are a heterogeneous mixture of particles, oxides of nitrogen, sulfur, carbon, dioxins furans, metals, chlorinated hydrocarbons (CHCs), and polycyclic aromatic hydrocarbons (PAHs). It is extremely challenging to understand the effect of potential synergisms between chemicals within the complex mixtures to which humans are exposed and delineate their potential health impacts.

We have reported the presence of environmentally persistent free radicals (EPFRs) associated with airborne fine and ultrafine PM samples collected from different locations across the United States [8-10]. We have further generated data demonstrating that the toxicity of real-world PM samples increases as a function of EPFR concentration (manuscript in preparation). The presence of EPFRs in real-world PM samples suggests a potentially unrecognized risk factor for the development and/or exacerbation of asthma. Thus, we have developed a model for understanding the health impacts of combustion-generated ultrafine particles (CGUFP) [11]. Specifically, we have developed CGUFP containing EPFRs using 1,2-dichlorobenzene (DCB230) and lacking EPFRs (DCB50) to understand their role in the development of asthma.

While exposure to PM causes adverse health effects in most people, children are especially susceptible to these effects, as they inhale more air per pound of body weight than adults; spend more time outdoors; and possess immature immune systems. Exposure to ambient air pollution is correlated with significant deficits in respiratory growth, leading to clinically important deficits in lung function in children [12]. The present investigations assessed the effects of EPFR-containing CGUFP on lung function in developing neonatal rat lungs.

## Results

### Neonatal DCB230 exposure resulted in acute airway dysfunction

**Figure 1:**
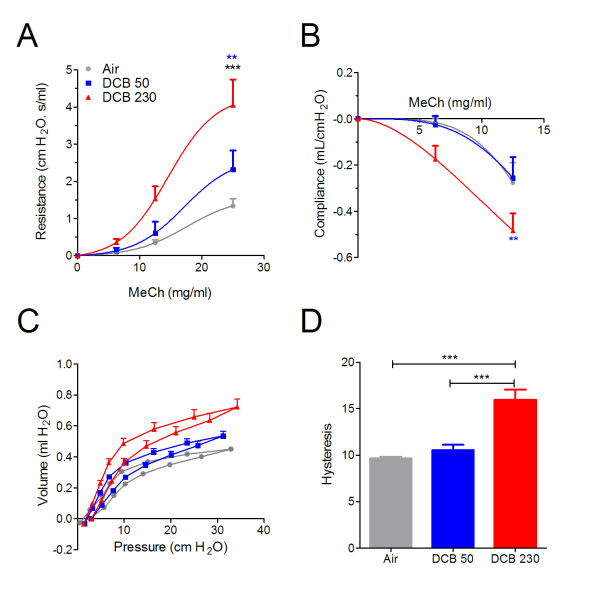
**Measurement of airway function following exposure to CGUFP**. Neonatal rats (7 d of age) were exposed to CGUFP for 20 m/d for 7 d and pulmonary function was assayed 24 hr after the final exposure. Significant increases in lung resistance (A) in response to MeCh challenge and decreases in compliance (B) among DCB230 exposed neonates compared with control groups (air and DCB50). Quasi-static inflation/deflation curves (i.e. pressure-volume loops) (C) and the area within the curves representative of hysteresis (D). Data are means ± SEM and is representative of three independent experiments with 6 animals/group. *p < 0.05, **p < 0.01, ***p < 0.001.

Neonatal rats were exposed to CGUFP at 200 μg/m^3 ^for 20 min/day for 7 consecutive days. Twenty-four hours after the final exposure, pulmonary function tests were performed on these animals. We compared the effects of EPFR-containing CGUFP (i.e. DCB230), the non-EPFR-containing CGUFP (i.e. DCB50), and ambient air on airway resistance in response to inhaled MeCh. We found that exposure to DCB230 significantly increased airway hyperreactivity (AHR; 4.1 ± 0.69 cm H_2_O.s/ml; Figure 1A) compared to the air-exposed control groups (Air: 1.3 ± 0.19). DCB50 did not significantly increase AHR (DCB50: 2.3 ± 0.51). There was a significant decrease in lung compliance (Figure 1B) among the DCB230 exposed rats compared to the controls (DCB230: -0.49 ± 0.075 ml/cm H_2_O vs. Air: -0.28 ± 0.09 or DCB50: -0.26 ± 0.09). Similarly, pressure-volume curve analysis illustrated a loss of lung static compliance in DCB230 exposed neonates (Figure 1C). The area within the quasi-static inflation/deflation curves, which represents hysteresis was also calculated (Figure 1D). The area for DCB230 lungs was significantly larger than air-exposed or DCB50 controls and there was no difference between air and DCB50 exposed animals.

### CGUFP enhances oxidative stress in neonatal lungs

**Figure 2:**
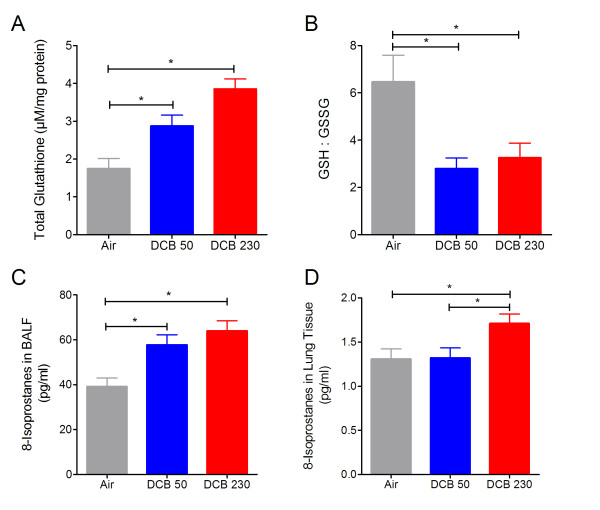
**Indicators of antioxidant status and oxidative stress in postnatal rat lungs following exposure to CGUFP**. Changes in total glutathione (A) and GSH:GSSG ratios (B) in whole lung homogenates and 8-Isoprostanes in BALF (C) and whole lung homogenates (D) after CGUFP exposure. All measurements were made 24 hr post-exposure. n = 4-6 animals/group. Data are means ± SEM. *p < 0.05.

Glutathione, a key antioxidant involved in maintaining proper lung redox balance, was measured to determine pulmonary oxidative stress after CGUFP exposure in neonates. DCB230 and DCB50 exposed neonates had significantly higher total glutathione levels than air exposed groups analyzed (Figure 2A). However, the GSH:GSSG ratios in DCB230 and DCB50 exposed neonates were significantly lower than that of the air exposed group (Figure 2B). No difference in GSH or GSH:GSSG ratios was observed between DCB50 and DCB230 exposed groups. Exposure to DCB230 or DCB50 resulted in elevated levels of 8-isoprostanes in the BALF (Figure 2C) compared to air exposed neonates. Elevated levels of 8-isoprostanes was evident only in the lungs from DCB230 exposed neonates (Figure 2D).

### DCB230 increased airway lymphocyte infiltration neonatal rats

**Figure 3:**
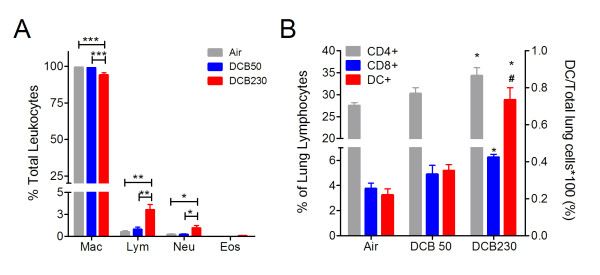
**Effects of CGUFP exposure on pulmonary inflammation**. (A) BALFs were collected and cell differentials obtained. *p < 0.05, **p < 0.01, and ***p < 0.001. (B) Lymphocyte populations in the lungs of rats exposed to CGUFP were quantified by flow cytometry using antibodies specific for the indicated cells after gating on lymphocytes (CD4 and CD8) or non-lymphocytes (DC). Mac indicates macrophages; Neu: neutrophils; Lym: lymphocytes; Eos: eosinophils. n = 4-6 animals/group. *p < 0.05 vs. air and #p < 0.05 vs DCB50.

The total number of leukocytes recovered in the BALF was significantly elevated in the DCB230 exposed animals. Marked increases in the number of lymphocytes and neutrophils recovered from the BALF occurred only with DCB230 exposure (Figure 3A). An increase in eosinophils in the BALF from DCB230 exposed animals was also observed; however, this number was not significantly different from the DCB50 or air exposed animals and represented less than 0.08% of the total recovered cells.

To begin to address the possible mechanisms responsible for DCB230 induced AHR, we assessed the relative contributions of lymphocyte subpopulations to the airway inflammation observed in CGUFP-exposed neonates. DCB230 exposure augmented the influx of both CD4+ and CD8+ lymphocytes and of dendritic cells into the lung compared to the controls (Figure 3B).

### Altered cytokines in neonatal lungs exposed to DCB230

**Figure 4:**
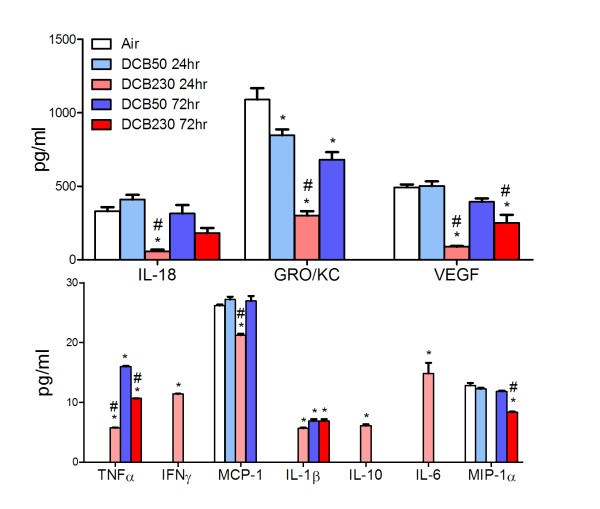
**BALF cytokine levels in postnatal rats after CGUFP exposure**. Cytokine levels were determined in the BALF supernatant at 24 hr and 72 hr post-final exposure. n = 6-12 animals/group. Data are expressed as means ± SEM. *p < 0.05 vs Air and #p < 0.05 vs day-matched DCB50.

Pulmonary inflammation in the air, DCB50, and DCB230 exposed animals was further quantified by measuring the levels of various cytokines in the BALF at 24 and 72 hr after the final exposure. The following cytokines were analyzed: IL-1β, IL-10, IL-18, IL-6, IFN-γ, TNF-α, GRO/KC, VEGF, MCP-1, and MIP-1α At 24 hr post-final exposure we observed decreases in IL-18, VEGF, MCP-1, and MIP-1α in DCB230-exposed animals compared air-exposed controls (Figure 4) while GRO/KC levels were decreased in both DCB50 and DCB230 exposed animals; this effect was more pronounced with DCB230 exposure. At the same time point, an increase in TNFα, IL-1β, IFNγ, IL-10, and IL-6 was observed only in DCB230 exposed groups. At 72 hr post-final exposure, IL-18, VEGF, and MIP-1α levels were raised to a similar level as air controls, though VEGF and MIP1α remained statistically different. GRO/KC levels continued to lower from the previous timepoint in both DCB50 and DCB230 animals. MCP-1 levels also continued to decline only in the DCB230 group. Previous spikes seen in IFNγ, IL-10, and IL-6 were diminished, while production of TNFα continued to increase in both DCB50 and DCB230 groups. Elevated levels of TNF-α at 24 hr post-final exposure were also increased at 72 hr compared to air controls. IL-1β was observed in DCB50 group and remained high in the DCB230 group at this time.

### DCB230 induces distinct changes in pulmonary architecture

**Figure 5:**
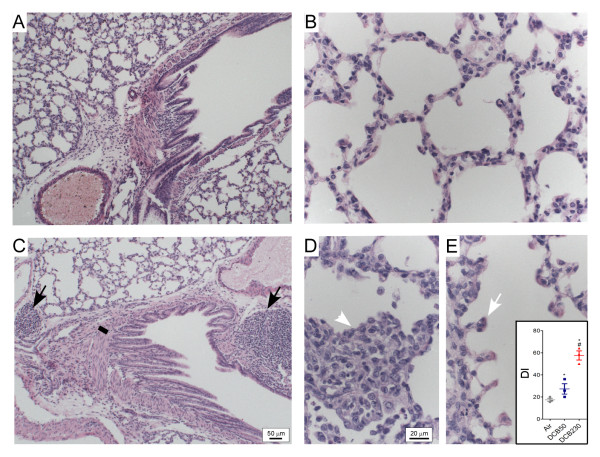
**Light micrographs of exposed rat lungs**. Light micrographs of terminal bronchioles (A, C) and alveolar parenchyma (B, D, E) from 15 d old rat lungs following exposure to DCB50, which was visually identical to air (A, B), and DCB230 (C-E). Black arrows denote significant peribronchiolar BALT; line denotes smooth muscle mass surrounding bronchiole (quantified in Figure 6); white arrow denotes lesions of increased alveolar space (quantified in inset of E); and white arrowhead demonstrates alveolar occlusion. Bar represents 50 μm (A, C) and 20 μm (B, D, E).

**Figure 6:**
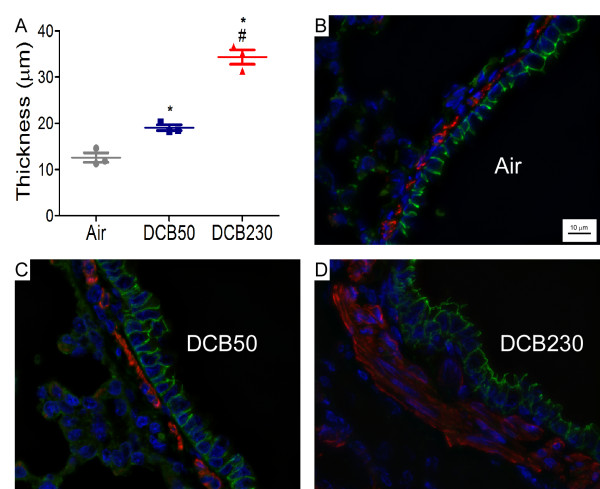
**Quantitation of peribronchiolar smooth muscle content in exposed rat lungs**. (A) Quantitative assessment of the thickness of the smooth muscle layer in major airways of 15 d old rat lungs after exposure to air, DCB50 or DCB230. Data represent means ± SEM. n = 3 animals/group. *p < 0.05 vs Air and #p < 0.05 vs DCB50. (B, C, D) Representative micrographs demonstrating expression of α-smooth muscle actin (red) and E-cadherin (green) from 15 d old rat lungs exposed to air, DCB50, or DCB230, respectively. Cell nuclei stained with DAPI (blue).

Normal histoarchitecture was observed in the lungs of the air and DCB50 exposed neonates (Figure 5A; DCB50 exposed lung is used as representative control). In contrast, neonates exposed to DCB230 exhibited significant lymphoid aggregates in the peribronchial region (Figure 5C). This was accompanied by areas of immense infiltration of macrophages resulting in occlusion in the alveolar spaces (Figure 5D) and distinct focal changes in alveolar structure including septal destruction (DI: Air: 17.35 ± 1.38% vs. DCB50: 27.31 ± 4.71 vs. DCB230: 57.6 ± 4.11; Figure 5E). Although quantitative morphological assessments were not performed, we also observed a consistent, subtle decrease in the thickness of the alveolar walls in neonatal rats exposed to DCB230 (Figure 5C). Further analysis of the H&E sections revealed what appeared to be an increase in the smooth muscle mass in the peribronchial regions of DCB230 exposed mice; therefore, sections were stained for α-smooth muscle actin (α-SMA) and morphometric analyses performed to quantitate α-SMA thickness. Animals exposed to DCB230 possessed significantly greater smooth muscle mass in the peribronchial region compared to animals exposed to either air or DCB50 (Air: 12.58 ± 1.03 μm vs. DCB50: 19.05 ± 0.63 vs. DCB230: 34.34 ± 2.71; Figure 6).

### CGUFP exposure and protein expression in lungs

**Figure 7:**
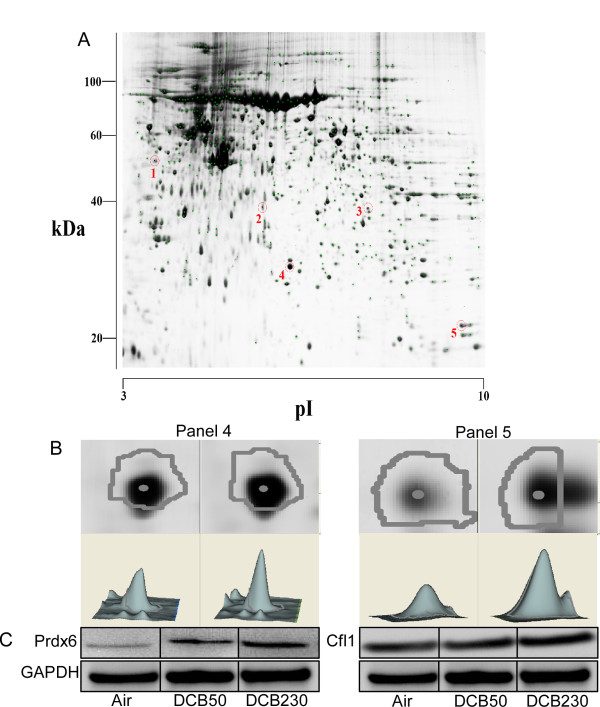
**Proteomic analysis of DCB230 exposed neonatal lungs**. (A) 2-D gel proteomic map with selected spots indicating a significant intensity change vs air exposed controls. (B) Enlargement of panels 4 and 5 from air and DCB230 exposed lungs. Panel 4 indicates PRDX6 expression (1.33 increase, p < 0.00034) and panel 5 indicates CFL1 expression (1.34 increase, p < 0.0008). (C) Western blot confirmation of increased expression of both proteins following exposure to DCB230 as compared to air or DCB50. n = 4-6 animals/group.

To address the mechanism of altered lung structure and function following DCB230 exposure in neonates, we isolated total protein from the lungs of DCB230, DCB50, and air exposed rats. The protein samples were separated by 2-dimensional electrophoresis, and the protein spots were visualized following fluorescent staining. Differential proteome maps, which were the overlaid gel images of air and DCB230; air and DCB50; and DCB50 and DCB230 treated groups, showed alterations in the expression of several protein spots following exposure. We identified 3 spots that fit our test criteria (i.e. having a t-test p value ≤0.05 and 30% fold change (-1.30 or 1.30)) in the DCB50 versus air comparison; 16 spots in the DCB230 versus air comparison, and 2 spots in the DCB50 versus DCB230 comparison. Two of the spots were found in both the DCB50 versus air comparison and in the DCB230 versus air comparison. All 12 spots of particular interest (i.e. greatest up- or down-regulation) were isolated from the appropriate gels and analyzed by MALDI-TOF-MS following in-gel digestion (Figure 7A). We were able to identify the proteins from 5 spots. DIGE analysis revealed a remarkable upregulation of cofilin-1 (CFL1) in the lungs of DCB230 exposed compared to air and DCB50 exposed neonatal rats (Figure 7B). DIGE also unveiled a slight upregulation of peroxiredoxin-6 (PRDX6) in the lungs of DCB50 exposed compared to air, and a significantly larger upregulation in DCB230 exposed compared to air. DIGE data were verified by western blot analysis (Figure 7C). Sulfotransferase 1A1 and annexin A8 expression were also increased in the DCB230 vs. air and DCB50 vs. air differential proteome maps. Creatine kinase M-type was downregulated in the DCB230 compared to air exposed neonatal rat lungs.

## Discussion

PM air pollutants exacerbate a variety of pulmonary disorders, including chronic obstructive pulmonary disease [13,14], asthma [15,16] and lower respiratory tract infections, especially in infants and the elderly [17-21]. The lungs of neonates and infants are undergoing tremendous structural and functional changes. Little information and only a few studies have been developed to understand the impact of exposure to environmental air pollutants during postnatal development.

Our results show that EPFR-containing DCB230 induced dramatic and sustained AHR and pulmonary inflammation in neonatal rats. The inflammation following DCB230 exposure was characterized by increased lymphocyte and neutrophil cells in the airways and by elevations in the cytokines TNF-α, IL-1β, IFNγ, IL-10, and IL-6 within 24 hr of exposure. Decreases in IL-18, GRO/KC, MCP-1, MIP-1α and VEGF were observed within this same time frame. By 72 hr after exposure, some cytokines (IL-18, VEGF, IFNγ, IL-10, IL-6, and MIP-1α) had begun to return to control levels while earlier trends seemed to persist for others (GRO/KC, TNFα, IL-1β). No changes in lung function were observed following exposure to either of the controls (air or non-EPFR-containing DCB50). The lung dysfunction observed following DCB230 exposure correlated with increased oxidative stress as indicated by elevated glutathione levels, decreased GSH:GSSG ratios, and increased 8-isoprostanes in the BALF and lungs of DCB230 exposed neonates. The increased AHR observed here in neonates is consistent with the findings of many investigators studying the impact of ambient PM on AHR in adult rodent exposure models [22-24].

Eosinophilic inflammation is a hallmark of allergic asthma and we found it somewhat surprising that increases in this cell population were not observed in response to DCB230 exposure in either the lavage fluid or the lung tissue. There are several potential reasons increases in eosinophils was not observed: 1) eosinophilic response to PM is strain/species specific; 2) the age of the animal at the time of exposure plays a role in the cellular response that develops [25,26]; and/or 3) the chemical composition of the particles is important in determining the cellular response. Eosinophils were observed following PM exposure in adult A/J mice, which are known to generate a strong eosinophilic response. Brown-Norway rats, which are also known to exhibit a strong allergic phenotype including elevations in pulmonary eosinophils in response to allergen exposure, were used in these studies and therefore, the absence of eosinophils does not appear to be a strain-specific response. We have demonstrated that age of initial exposure to certain viruses is critical in determining the ensuing cellular and immune response. We are currently performing equivalent exposures to DCB230 in adult animals to determine if the lack of eosinophils is an age-specific phenomenon. Finally, composition of the particles is also likely to play a significant role in defining the type of inflammation initiated and the ensuing T cell response and may thus account for the observed difference in eosinophil levels.

Interestingly, we observed an increase in neutrophils in the lavage fluid. An influx of neutrophils has been reported following adult murine exposure to ambient PM [22,24,27] and is observed in as many as 50% of all asthmatics and correlates with asthma severity [28-30]. In contrast to the increase in neutrophils, we observed a significant decrease in macrophages in the lavage fluid at 24 hr post-exposure. Although studies are ongoing to fully decipher the reason for this decrease in macrophage/monocyte cells from the lavage fluid, it appears that these cells along with DCs are the principal cells responsible for particle uptake in the lung and that there is enhanced recruitment of these cells to the draining lymph node following exposure (manuscript in preparation). Interestingly, an adaptive immune response as evidenced by increased CD4+ and CD8+ T lymphocytes was also observed following exposure to DCB230. It is assumed that the lymphocytes are recruited in response to chemokines secreted by antigen presenting cells and that their role is to provide protection against subsequent exposures to DCB230. Furthermore, these cells most likely play a significant role in both the observed AHR and pulmonary pathology following DCB230 exposure. Future studies are being designed to determine how these cells are recruited to the lung as well as their role in the observed pulmonary pathophysiology associated with EPFR exposure.

Several pieces of data support the involvement of oxidative stress in EPFR-induced pulmonary inflammation and injury in this neonatal exposure model. First, we saw increased production of 8-isoprostanes, a marker of the oxidation of tissue phospholipids, in the lungs and lavage fluid of exposed neonates. Oxidative stress was most prominent in neonates that had been exposed to DCB230. Augmented levels of isoprostanes have been found in serum, plasma and urine of heavy smokers [31,32] and in exhaled condensate of asthmatic patients [33]. Second, we observed an increase in total glutathione that correlated with a decreased ratio of GSH:GSSG indicative of oxidative stress. Increased oxidative stress has been observed in the epithelial lung fluid of children with severe asthma [34] and is associated with pulmonary deterioration [35,36]. Decreased GSH:GSSG ratios in conjunction with increased 8-isoprostane levels in the lungs seem to confirm a role for EPFR induced lung injury in neonatal rats and also offers a valid mechanism by which exposure to PM exacerbates allergic asthma.

Exposure to DCB50, a non-EPFR containing particle, also induced some amount of oxidative stress. In these animals, 8-isoprostane levels in the lung tissue were significantly lower than that observed following DCB230 exposure and no significant changes in lung function were observed following exposure to these particles. From our previously published data, we believe this is due to the enhanced ability of EPFRs to modulate the activity of a number of antioxidant enzymes including catalase and glutathione reductase [37]. Cumulatively, these data suggest that EPFRs play a significant role in the adverse pulmonary responses observed and that oxidative stress is only partly responsible for the observed pulmonary responses.

We employed DIGE-based protein profiling to discern the effects of CGUFP on protein expression in the lungs of exposed neonates. Two of the proteins identified by differential DIGE analysis and validated by western blotting can be linked to a possible sequence of events that begin with a pro-oxidative effect initiated by the EPFRs and culminating with the increased expression of a protein that may be important in the regulation of glucocorticoid sensitivity of T lymphocytes and steroid-insensitive asthma. Those two proteins are PRDX6 and CFL1.

PRDX6 is believed to play an important role in catalyzing the reduction of peroxides via its anti-oxidative activity, which involves removal of H_2_O_2 _in primary lung alveolar epithelial type II [38] and other cells [39] and inhibition of LDL oxidation by reducing the highly reactive hydroxyl radicals (OH^•^) and HOCl [39]. Previous studies with EPFR-containing particles demonstrate that EPFRs are a potent stimulator of LPO in vitro [40] and that these inductive events contribute to EPFR-induced pathologies. Further, PRDX6 can emulate glutathione peroxidase [41] and inhibit the expression of intercellular adhesion molecule-1 and vascular cell adhesion molecule-1, both of which help recruit macrophages. These observations support the hypothesis that PRDX6 plays a key role in protecting lung tissue from oxidative stress injury (due to peroxides) caused by CGUFPs including EPFRs.

We also observed an increase in CFL1, which has been implicated as an inhibitor of glucocorticoid function in hormone-resistant HeLa cells and in CD4+ T cells from patients with severe, glucocorticoid-insensitive asthma [42-44]. The increase in CFL1 expression in the lungs of neonatal rats exposed to EPFR-containing particles suggests that pediatric exposure to EPFR-containing PM is a potential mechanism responsible for the development of severe, glucocorticoid-insensitive asthma in humans. Ongoing studies in our laboratory seek to confirm the role of CFL1 in dexamethasone insensitivity following exposure to EPFRs.

On the whole, our findings suggest that EPFRs have the potential to induce oxidative stress in the lungs of neonates following acute inhalation exposures and that cytoprotective responses are initiated to deal with this stress (i.e. PRDX6) to protect the developing lung. However, the ability to control the pro-oxidant effects of EPFRs eventually overwhelms the system leading to the increased expression of CFL1 [45]. Active CFL1 then reduces nuclear translocation of glucocorticoid receptors allowing for enhanced proinflammatory transcription [46]. Further studies are needed to confirm the hypotheses generated by these studies, but our data demonstrate a potential mechanistic link between PM exposure and the development of asthma in humans.

## Conclusions

On the whole, proteome profiles as well as all the associated cellular responses posits our hypothesis that EPFRs contained in CGUFP have the potency to induce oxidative stress leading to the induction of pulmonary inflammation and dysfunction. Since the formation of EPFRs is linked to a chemical reaction with transition metals contained in ultrafine particles, it is important to realize that the particles must be considered an EPFR-transition metal-substrate system. Separation and independent study of these components is likely to lead to erroneous conclusions concerning the toxicity of environmental PM.

Overall, there are several factors that contribute to the lung injury in neonates following exposure to CGUFP. Data presented in this article suggest that exposure to EPFR-containing CGUFP is one of the major factors influencing the inflammatory and pathophysiological responses. Concerns are to understand whether the changes caused by these exposures are reversible, and the role of long-term exposures in the development of steroid-insensitive asthma in humans.

## Methods

### Rats

Brown-Norway rat dams and pups were procured from Harlan (IN, USA). Animals were maintained in ventilated cages housed in a specific, pathogen-free animal facility. Animal protocols were prepared in accordance with the Guide for the Care and Use of Laboratory Animals and approved by the Institutional Animal Care and Use Committee at Louisiana State University Health Sciences Center.

### Combustion Generated Ultrafine Particles (CGUFP)

Particles were synthesized essentially as previously described and had a mean diameter of 0.2 μm [11]. In brief, EPFR containing CGUFP (DCB230) was formed as follows: 5% CuO supported on silica was dosed with adsorbate vapors (i.e. 1,2 dichlorobenzene) in a custom made vacuum exposure system for 5 min at 10 torr at 230°C. Any physisorbed dosant was removed by evacuation at 10^-2 ^torr, and the particles were allowed to cool to room temperature under vacuum. The presence of free radicals was confirmed by electron paramagnetic resonance. Based on previous studies [11] it is known, that 1,2-dichlorobenzene adsorbed on CuO/Silica particles at 230°C forms primarily *o*-semiquinone EPFRs. Non-EPFR containing CGUFP (DCB50) was formed by dosing silica with adsorbate vapors (i.e. 1,2 dichlorobenzene) in a custom made vacuum exposure system for 5 min at 10 torr at 50°C. The particles were allowed to cool to room temperature and evacuated at 10^-2 ^torr to remove remaining gas phase reactant, which allowed for the formation of particles containing silica matrix and adsorbed molecular 1,2-dichlorobenzene as reference material to 1,2-dichlorobenzene originating EPFRs. The absence of free radicals was confirmed by electron paramagnetic resonance. The size of the particles was confirmed prior to experiments using flow cytometry also as previously described [40].

### Animal Exposures

Neonates (7 days of age) were divided into three exposure groups: Air, DCB50, and DCB230. Neonates were subjected to nose-only exposures of DCB230 or DCB50 at 200 μg/m^3 ^or air using the In-expose inhalation system (SciReq) for 20 min/day for seven consecutive days. All assessments were performed 24 hours following the final exposure. Cytokines were additionally assessed at 72 hours following the final inhalation exposure. Each group consisted of 4-6 rats, and all experiments were performed in triplicate.

### Pulmonary Mechanics

Respiratory mechanics were measured using the forced oscillation technique (FlexiVent; Scireq) as previously described [25]. Exposed rats were anesthetized, intubated, and mechanically ventilated by a computer controlled piston ventilator. Rats were then challenged with an aerosolized bronchoconstrictor, methacholine (MeCh), at increasing doses (MeCh: 0, 6.25, 12.5, 25 mg/ml). At each dose, lung resistance, compliance, and elastance were calculated using the single compartment model and pressure-volume data were captured using a step-wise quasi-static inflation/deflation maneuver to total lung capacity/functional residual capacity, respectively. For comparison among the groups and across measurement days, all data were normalized to their individual baseline resistance values ((value-baseline)/baseline) and plotted as normalized resistance. Baseline values ranged from 0.701 to 0.805 cm H_2_O·s/ml and were not statistically different among the groups.

### Glutathione Levels

Briefly, tissue was homogenized in 5% sulfosalicylic acid (in ice-cold 5% metaphosphoric acid mixture) and centrifuged at 2500 g for 10 min, 4°C. An aliquot of the supernatant was used for the assay. Oxidation of GSH by 5,5'-dithio-bis (2-nitrobenzoic acid) to form nitrobenzoic acid and the enzymatic recycling of glutathione (GSH) from glutathione disulfide (GSSG) by glutathione reductase (GR) in the presence of NADPH were spectrocphotometrically measured at 412 nm [47].

### 8-Isoprostanes

Lipid peroxidation was measured by a competitive enzyme-linked immunosorbent assay (ELISA) for 8-iso-PGF with a commercial kit (Cayman Chemical, Ann Arbor, MI). The assay is based on the competition between 8-iso-PGF and 8-isoprostane-acetylcholinestase (AChE) conjugate for a limited number of binding sites in each ELISA plate well. The concentration of 8-iso-PGF is inversely proportional to the number of binding sites available, whereas AChE is held constant. For lung homogenates, samples were weighed and homogenized in 0.1 M PBS (1 mM EDTA, 0.005% butylated hydroxytoluene). Samples (BALF and lung homogenates) were transferred to the ELISA plate and incubated with the antibody for 18 hr. The absorbance of the colorimetric enzymatic reaction was read at 405 nm using the SpetraMax-M2 (Molecular Devices, Sunnyvale, CA) and compared with an 8-iso-PGF standard curve to calculate concentration.

### BALF Cellularity

Bronchoalveolar lavage fluid (BALF) was harvested in 1 ml of PBS containing 2% BSA. Isolated BALF was used to determine total number of leukocytes. Cytospin slide preparations were made, stained with Diff-Quik (Fisher Scientific), and used for differential cell counts. All counts were performed by two unbiased observers using standard morphological criteria to classify individual leukocyte populations. Six rats from each group were used for these analyses, and 200 cells were counted per animal.

### Pulmonary Lymphocyte Characterization

A single cell suspension of lung cells was prepared using a standardized protocol [48]. Briefly, lungs were perfused, excised, cut into small pieces and incubated at 37°C for 1 hour in RPMI-1640 media supplemented by 2% heat inactivated FBS, 1 mg/ml collagenase I (Invitrogen), and 150 μg/ml DNase I (Sigma). After incubation, single cells were obtained by mashing the lung pieces through a 40 μm cell strainer (BD Biosciences). Red blood cells were lysed using 1× RBC lysis buffer (eBioscience) and cells were stained with the following antibodies purchased from BD Pharmingen: APC-CD4, FITC-CD8, and Biotin-CD3, and from Santa Cruz: PE-OX62. Cell staining was determined with an LSRII (BD Biosciences) flow cytometer after gating on the lymphocyte population as determined by forward and side scatter properties. Flow data were analyzed and plotted using FlowJo software (Version 7.2.2 for windows, Tree Star, Inc).

### Cytokine Levels in BALF

Cytokine levels were measured from 50 μl of cell-free BALF (rat cytokine assay; Millipore, MO, USA) using a high-throughput multiplex cytokine assay system according to the manufacturer's instructions. Each sample was analyzed in duplicate on the Bio-Plex 200 system (BioRad). A broad range of standards (4.88 to 20,000 pg/ml; depending on the analyte) was used to quantitate a dynamic range of cytokine concentrations. The concentrations of analytes in these assays were quantified using a standard curve and a 5-parameter logistic regression was performed to derive an equation that was then used to predict the concentration of the unknown samples. The following cytokines were assayed: IL-1β, IL-10, IL-18, IFN-γ, TNF-α, GRO/KC, VEGF, MCP-1, and MIP-1α. The data presented excluded any value outside the range of sensitivity for the particular analyte.

### Pulmonary Histopathology

Lungs were perfused with PBS and heparin, inflated by gentle infusion of HistoChoice Tissue Fixative (Amresco, Inc.) to total lung capacity, and isolated. The fixed lungs were then dehydrated, embedded in paraffin, and sectioned at 4 μm sections. Each lung section was stained with hematoxylin and eosin (H&E). Lung sections were probed for E-cadherin and α-smooth muscle actin (SMA) using the following antibodies (Abcam) and dilutions: anti-SMA (1:1000), E-cadherin (1:400). Primary antibodies were respectively detected with goat anti-mouse Alexa Fluor 568 (1:500) and with Goat anti-Rabbit Alexa Fluor 488 (1:500) both from Invitrogen. Cell nuclei were stained with DAPI.

Alveolar septal lesioning was quantified from H&E stained lung sections of animals exposed to air, DCB50, and DCB230. The destruction of alveolar walls was quantified using the destructive index (DI) method [49]. A grid with 42 points that were at the center of hairline crosses was superimposed on the lung field. Structures lying under these points were classified as normal (N) or destroyed (D) alveolar and/or duct spaces. Points falling over other structures, such as duct walls, alveolar walls, etc. did not enter into the calculations. The DI was calculated using the formula: DI = D/(D + N) × 100.

To quantify smooth muscle thickness, images of major airways were taken at 40× magnification from lung sections of rats exposed to DCB230, DCB50, or air. Using AxioVision software, a line perpendicular to the basal lamina was drawn from the basement membrane to the edge of the smooth muscle layer. Smooth muscle cells were identified according to morphology and stain. After this was repeated 4-5× per airway, an average was taken from the software-generated thickness values (μm). Averages from 3 different animals per exposure group were compared.

### 2D-Gel Electrophoresis and Mass Spectroscopy to Identify Proteins

#### Tissue Preparation and Protein quantification

Lungs from exposed animals (4 animals/group) were sonicated following the addition of 400 μl of Lysis Buffer (7 M Urea, 2 M ThioUrea, 4% CHAPS, 30 mM Tris, pH8.5, and 20% glycerol). Sonication was performed 4 times at 25% amplitude for 15 seconds each; returning to ice in between. Protein concentrations were determined by Bradford protein assay using an 8 point BSA standard concentration curve. For CyDye labeling, samples were aliquotted into 50 μg fractions.

#### Cy-Dye labeling

The mixed internal standard methodology of [50] was used in these studies. Briefly, aliquots of 50 μg protein from each sample were labeled with 400 pmol of either Cy3 or Cy5 (vehicle and/or treated randomized). In a similar fashion, 50 μg of each of the samples (vehicle and treated) was pooled and labeled with 400 pmol Cy2 per 50 μg standard. Equal protein loading of replicate #1 versus replicate #2, and the standard-sample mixture was resolved between pH 3-10NL per gel. Samples were dissolved in rehydration buffer (7 M Urea, 2 M ThioUrea, 4% CHAPS, 20% glycerol) supplemented with IPG buffer (GE Healthcare).

#### 2-D gel electrophoresis and imaging

Cy-dye labeled samples for each subject (410 μl final volume) were actively rehydrated into 24 cm 3-10NL immobilized pH gradient (IPG) strips (GE Healthcare) for 15 hours, followed by isoelectric focusing using an IPGphor (GE Healthcare) step 1: Step 300 V for 2 hours, step 2: Gradient 1000 V for 6 hours, step3: Gradient 8000 V for 6 hours, step 4: Step 8000 V for 8 hours, and step 5: step 200 V for HOLD. The cysteines were reduced and carbamidomethylated, while the proteins were equilibrated into the second-dimensional loading buffer by incubating the focused strips in equilibration buffer (6 M Urea, 20% glycerol, 2% SDS, 375 mM Tris, pH8.8) supplemented with 20 mg/ml DTT for 15 min at room temperature with shaking, followed by 25 mg/ml iodoacetamide in equilibration buffer for an additional 15 min room temperature incubation. IPG strips were then cemented onto 2^nd ^dimension gels using an overlay consisting of 0.5% agarose in SDS running buffer (25 mM Tris, 192 mM glycine, 0.1% SDS, trace of bromophenol blue). Homogeneous polyacrylamide gels (12%) were used for the second-dimensional SDS-PAGE which was then carried out on all gels simultaneously using a DALT6 (GE Healthcare) at 5 W/gel for 30 min followed by 17 W/gel for 4 h. The Cy2 (standard), Cy3 and Cy5 (vehicle or treated) for each gel were individually imaged using mutually exclusive excitation/emission wavelengths of 488 nm (ex) and 520 nm (em) BP40 (bandpass) for Cy2, 532 nm (ex) and 580 nm (em) BP 30 for Cy3, and 633 nm (ex) and 670 nm (em) BP30 for Cy5 using a Typhoon 9400(GE Healthcare). After imaging for Cy-Dye components, the non-silanized glass plate was removed, and the gels were fixed in 10% methanol, 7% acetic acid for 1 h, rinsed in water three times and then incubated in SYPRO Ruby in the dark overnight. The SYPRO Ruby post-stain allows for the correction of unlabeled protein's migration in relation to the 1-3% CyDye labeled migration, and ensures accurate protein excision. Sypro Ruby images were acquired on the same imager using 450 nm (ex) and 610 nm BP40 filter, as well as re-imaged post-excision to ensure accurate protein excision.

#### DIGE analysis

DeCyder software (GE Healthcare) was used for simultaneous comparison of abundance changes across all samples, and for comparisons of individual Cy3 and Cy5 samples for each subject. Difference ratios or abundance changes and paired Student's *t*-test *p*-values for the variance of these ratios for each protein pair across all samples were calculated. Fold abundance changes are reported, whereby a fold increase is calculated directly from the volume ratio and a fold decrease by the inverse of volume ratio.

#### In-gel digestion

Proteins of interest were excised using the Ettan Spot Picker (GE Healthcare) based on a 'hit list' generated in DeCyder. Spots were de-stained by successive changes of 20 mM ammonium bicarbonate and 50% acetonitrile, followed by dehydration with a 20 minute incubation with 100% acetonitrile. Dehydrated gel plugs were automatically digested in-gel with 8 μL 20 μg/ml porcine modified trypsin protease (Promega) in 20 mM ammonium bicarbonate for 6 hours at 37°C. Tryptic peptides were then extracted from the gel plugs in two cycles of 50% acetonitrile, 0.1% trifluoroacetic acid and dried by evaporation. Peptides were reconstituted in 1 μl 50% Acetonitrile and 0.1% trifluoroacetic acid and mixed with an equal volume of 10 mg/ml α-Cyano-4-hydroxy-cinnamic acid for spotting onto a MALDI plate.

#### Mass Spectrometry and Identification of Proteins

Matrix-assisted laser-desorption ionization time-of-flight mass spectrometry (MALDI-TOF-MS): The parent polypeptides were identified by comparing the profile of tryptic peptide masses generated by the mass spectrometer with predicted tryptic peptides from all known polypeptides using the MASCOT program. Since a covalent modification such as thiolation or nitration changes a peptide mass by a known amount, it is possible through MASCOT and other programs to identify both a protein and its posttranslational modifications.

### Statistical Analysis

All data were plotted as mean ± SEM and analyzed using GraphPad Prism (GraphPad Software Inc., Version 5.0.0). Two-way ANOVA (Bonferroni post-test) was used to evaluate the differences of airway responsiveness, airway resistance, elastance, compliance, and BALF cellularity. One-way ANOVA was used to evaluate the differences of PV loop, cytokine levels between groups, alveolar septal destruction index, and smooth muscle proliferation. Tukey's one-way analysis of variance was performed to test for significance between the groups. Differences between means were considered significant when p < 0.05.

## Abbreviations

CGUFP: combustion generated ultrafine particles; COPD: chronic obstructive pulmonary disease; DCB50: non-EPFR particle consisting of 1,2-dichlorobenzene physisorbed to silica/CuO; DCB230: EPFRs of 1,2-dichlorobenzene chemisorbed to silica/CuO at 230°C; EPFRs: environmentally persistent free radical-containing pollutant-particle systems; PM: ultrafine particulate matter; ROS: reactive oxygen species

## Competing interests

The authors declare that they have no competing interests.

## Authors' contributions

SB and SC: Conceived the study, participated in its design and coordination and drafted the manuscript. JS: Performed the experiments including pulmonary function testing, western blot analysis, and cytokine assays and participated in drafting the manuscript. PT: Performed IHC imaging analysis and assisted with smooth muscle mass quantification. TA: assisted with exposures and pulmonary function testing. SL and BD: Created and characterized the CGUFP particles. All authors have read and revised the manuscript critically for important intellectual content and have given final approval of the version to be published.
